# Egyptian School of Medicine

**Published:** 1840

**Authors:** 


### REVIVAL OF MEDICAL INSTRUCTION IN EGYPT.

Egypt seems to have been the birth place of medicine and her priests the first authorized prescribers. If they departed from the rules, derived from Osiris, or some other god, and the patient died, capital punishment was the consequence. No improvement was, of course, made under this system. Long afterwards, a different kind of medical science found its way into Alexandria, where many Greek and Arabic teachers of considerable distinction, made that famous city an emporium of the sciences. In the progress of time, this second epoch passed away, and a long night of ignorance and barbarism followed. The nineteenth century has seen this obscuration dissipated, and a third era commence. Both the science and the professors, are now from the west of Europe, and we may anticipate for Egyptian science much more than a mere revival. The present century will surpass the brightest Arabic epoch, for Mohammed Alee will soon have a better school, than the Ptolemies ever erected. The following notice of this new experiment, from

*Wilde's Yachting Expedition along the shores of the Mediterranean*, will be read with interest, by all who have perused the ancient history of our profession. D.

"*The Pacha's College and School of Medicine.*—I was next transferred to the care of Dr. Sicher, who conducted me through the college and school of medicine, which, as I before stated, forms a part of the building of the hospital, so that the student has but to cross the court from his dormitory to the ward, and can proceed from thence in a few minutes to the dissecting theatre or lecture-room, become acquainted with materia medica under the same roof in which he sleeps, and enjoy his morning's walk in the botanic garden beneath his window. Besides this, they are all required to become acquainted with practical operative chemistry; and for that purpose are sent for a certain time to work at the chloride of lime and saltpetre manufactories. This system, added to that of the general medical education here given, is one well worthy of imitation in Great Britain, and reflects no small credit on its founder, Clot Bey.

"At the date of my visit there were three hundred students in the college, who were fed, clothed, educated, and *paid* by the Basha. The dormitories and other apartments of these young men were clean and airy, and they themselves appeared orderly and attentive. They all wear a uniform, are regularly drilled as soldiers, and rise in rank and pay according to their proficiency. The pay varies from twenty to fifty piasters a month; and they are allowed out of the college once a week on the Sabbath.

"The nominal duration of study is five years; but the greater number are drafted off into the army or navy after three years: some few remain as long as seven.

"The school of medicine consists of seven professorships, viz: anatomy and physiology, surgery, pathology and internal clinique, pathology and external clinique, medicine and chemistry, botany and materia medica, and pharmacy. Instruction is given by means of an Arab interpreter or dragoman; the professor writes his lecture, and it is translated to the class by the interpreter. The majority of the professors are French, and the salary is somewhat more than £200 a year. They are all obliged to wear the Egyptian uniform and shave the head, but no sacrifice of religion or principle is demanded; and I need hardly remark that all Europeans, or Christians, are under the protection of their respective flags; and, should they be convicted of any misdemeanour, must be handed over to their Consul.

"The laboratory contained a good chemical apparatus, and the dissecting-room several subjects. This latter indispensable requisite to medical education it would be scarcely worth mentioning, but that it occurred among a people whose strong religious prejudices prohibited even the touching of a dead body in some cases, and the introduction of this novel science was one of the most difficult things Mohammad Alee had to enforce for a long time. He in the first place referred it to the priesthood, who obstinately set their faces against it, declaring it utter incompatible with the religion of the Prophet of Mekka. The Basha's answer, that it was his royal wish and pleasure that they should legalize the act, and that, if they did not speedily do so, it was more than probable they themselves should form material for the first experiment in this branch of the practical sciences, soon brought them to reconcile their prejudices with his unbending will."

